# COVID-PIRO (Predisposition, Insult, Response, Organ Dysfunction) Score: A Reliable Predictor of Outcomes in COVID-19 Patients Admitted in Intensive Care Unit

**DOI:** 10.7759/cureus.18960

**Published:** 2021-10-21

**Authors:** Sunil Kumar, Sameera Dronamraju, Sourya Acharya, Praraj Jaiswal, Vidyashree Hulkoti, Dhruv Talwar, Sanyukta Hepat, Irhsad VS, Divit Shah, Jahnabi Bhagawati

**Affiliations:** 1 Department of Medicine, Jawaharlal Nehru Medical College, Datta Meghe Institute of Medical Sciences (Deemed to be University), Wardha, IND

**Keywords:** rural hospital, outcome, intensive care unit, piro score, covid-19

## Abstract

Introduction

To measure the severity of sepsis and pneumonia in adult patients with coronavirus disease 2019 (COVID-19), the PIRO model (predisposition, insult, response, organ dysfunction) was adopted as a scoring system. In this study, the PIRO model was modified to classify the severity of pneumonia in adults and predict mortality risk infected with severe acute respiratory syndrome coronavirus 2 (SARS-CoV-2), admitted to a tertiary intensive care unit (ICU) in central rural India.

Method

This prospective, observational study was conducted in the Department of Medicine, in rural medical college at Wardha, Maharashtra, India from May 2020 to May 2021. Patients with reverse transcription-polymerase chain reaction (RT-PCR) positive for COVID-19 and whose age was more than 18 years admitted in the intensive care unit were included in the study.

Results

A total of 240 patients were included in the analysis having mean age of 60.27 ± 15.3 years. Number of deaths were 115 out of 240 (48.3%). Mean ICU stay was 9.09 ± 6.34 days. PIRO score ≤14.5 had a mortality rate of 1.25% as compared to the group having PIRO>14.5 which had mortality of 27.5%, with a cure rate of 26.25% and 5% respectively in both groups (p = 0.0001).

Conclusion

COVID-PIRO modified PIRO score was a highly sensitive and specific model in predicting in-hospital mortality but it is moderately sensitive in predicting ICU stay.

## Introduction

Coronavirus disease 2019 (COVID-19), caused by severe acute respiratory syndrome coronavirus 2 (SARS-CoV-2) virus was first detected in late December 2019 in Wuhan, China, and quickly spread around the world, reaching pandemic proportions. The disease has been diagnosed globally in more than two hundred million people, resulting in the death of more than four million people worldwide [1,2]. This pandemic caused a havoc to global health in all countries. Healthcare systems in all countries face problems with higher requirements for critical care resources. To fulfill these requirements and for effective management of the pandemic, predictive models for prognosis are necessary. Several models have been applied in critically ill COVID-19 patients for the past two years [3].

Scoring systems for use in intensive care unit patients have been introduced and developed over the last 30 years. They allow an assessment of the severity of disease and provide an estimate of in-hospital mortality [4,5].

Lisboa and colleagues reported that a four-variable PIRO score calculated at the time of ventilator-associated pneumonia (VAP) onset and reflecting tertiles of risk correlated well with both mortality risk and need for resource use [6]. Rubulotta et al found an independent mortality contribution associated with variables reflecting each of the four PIRO domains and reported that an aggregate model based on these was highly predictive of hospital mortality [7]. Based on the literature models developed to predict lethal courses for one type of viral pneumonia may be applied to COVID-19 patients also.

In literature, very few studies are available in this regard. Hence the present study was planned to study the efficacy of PIRO score (COVID PIRO scoring) in predicting the mortality and ICU stay in intensive care unit patients of COVID-19.

## Materials and methods

This prospective, observational study was conducted in the Department of Medicine, Datta Meghe Institute of Medical Sciences, Wardha, Maharashtra, India from May 2020 to May 2021. Inclusion criteria were COVID-19 patients (positive by RT-PCR) aged 18 years or above, admitted to the ICU. Exclusion criteria were patients with primary diagnosis other than COVID-19, pregnant females and patients not willing to give informed consent.

**Figure 1:**
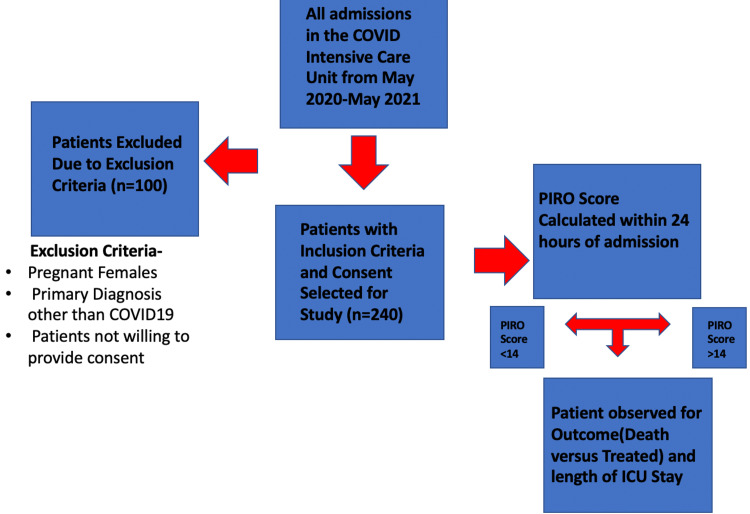
Flowchart showing the scheme of study.

Ethical approval for the study was obtained from the Institutional Ethics Committee of Datta Meghe Institute of Medical Science with approval number -Datta Meghe Institute of Medical Science (Deemed to be University)/Institutional Ethics Committee/May 2020-21/90 [DMIMS(DU)/IEC/May 2020-21/90]. A total of 350 patients admitted to the COVID ICU were considered for this study. After applying exclusion criteria a total of 240 patients meeting the inclusion criteria were included in the study after obtaining proper informed consent. All the COVID-PIRO score was calculated for each patient on arrival to within 24 hours of ICU admission. COVID-PIRO score was drafted after referring to the PIRO Score used previously for prognostication in cases of sepsis admitted in intensive care unit along with the clinical experience of the clinicians treating COVID19 in our centre and one faculty from Radiodiagnosis Department of Datta Meghe Institute of Medical Sciences who was kept blinded to the study aims and outcomes [8]. The different variables which were considered for the development of COVID-PIRO staging are given in Table 1. Flowchart depicting the study flow has been shown in Figure 1.

**Table 1:** COVID-PIRO score. Comorbidities: chronic obstructive pulmonary disease, diabetes mellitus, hypertension, obesity, ischemic heart disease. Inflammatory markers: D-dimer, (normal range-< or =500 ng/mL fibrinogen-equivalent units ), C-reactive protein (normal range-8-10 mg/L), interleukin 6 (normal range-less than 7pg/ml), serum lactate dehydrogenase (normal range-140-280 U/L), serum ferritin (normal range-24 to 336 micrograms per liter). Minimum score = 1 + 1 + 0 + 0 = 2. Maximum score = 4 + 6 + 9 + 1 = 21.

Predisposition (P)	Insult (I)	Response (R)	Organ Failure (O)
1) Age	1) Computed tomography score	1) Heart rate	Multiple organ dysfunction syndrome
20 to 40 -1 Score	< 8 - 1 Score	>120 beats/minute -3 Score	Present - 1 Score
40 to 60- 2 Score	9 to 15 - 2 Score		Absent - 0 Score
> 60 - 3 Score	> 15 -3 Score		
2) Comorbidity	2) Reverse transcriptase -polymerase chain reaction (cycle threshold length)-	2) Respiratory rate (with oxygen therapy)	2) Hypoxia
(any one) - 1 Score	>35 -0 Score	> 20 breaths/minute - 3 Score	PaO2 < 60 cm H2O - 1 Score
No comorbidity - 0 Score	35 to 25 -1 Score		PaO2 > 60 cm H 2O -0 Score
	< 25 - 2 Score		
	3) Parameter of inflammation	3) Hypotension	
	Any one parameter present - 1 Score	Systolic blood pressure-<90 mmHg - 3 Score	
	If Nil - 0 Score		

25-Point Computed Tomography Severity Score was used as it has been a useful Imaging tool for assessing severe COVID-19 [9]. Cycle threshold for RT-PCR was also noted as it has shown co-relation with viral load in various studies [10]. All enrolled patients were screened for co-morbidities on the basis of detailed history taking about any known previous illness and screening for various diseases including chronic obstructive pulmonary disease on the basis of an chest X-ray, ischemic heart disease based on electrocardiography, diabetes mellitus based on HbA1c, obesity on the basis of body mass index and hypertension on the basis of blood pressure all recorded within 24 hours of admission. Multiple organ dysfunction was diagnosed as impaired functioning of two systems of the body along with any two of the following: (a) temperature greater than 38.0°C or less than 36.0°C; (b) heart rate higher than 90 beats/min; (c) respiratory rate higher than 20 breaths/min or arterial carbon dioxide tension below 32 mmHg and (d) white blood cell (WBC) count higher than 12,000/µL, lower than 4000/µL, or including more than 10% bands.

All the patients were observed until cure or death and the length of hospital stay was also noted. Primary outcomes of the study were the use of COVID-PIRO score in the prediction of mortality as well as ICU stay. Secondary outcomes were correlation of co-morbidities with COVID-PIRO Score.

Laboratory measurements

RT-PCR for SARS-CoV-2 was used to diagnose COVID19 in the throat as well as nasal swabs. Central Clinical Laboratory of Jawaharlal Nehru Medical College was responsible for the diagnosis of COVID19 in all 240 patients enrolled in the study. The laboratory has been authorised by the Indian Council of Medical Research for conducting RT-PCR in order to diagnose COVID-19. Machine used for reverse transcriptase polymerase chain reaction was RT-PCR Quant Studio 5 which works on the principle of reverse transcription of ribonucleic acid to deoxyribonucleic acid for amplification. Kit used for RT-PCR was Meril COVID-19 One-Step RT-PCR kit which contained COVID-19 enzyme mix (lyophilized), COVID-19 primer-probe mix, enzyme mix buffer, COVID-19 PCR positive control and COVID19 negative control.

Interleukin 6 levels were measured using Robonic Washwell Plate ELISA Washer and Robonic readwell touch ELISA Plate analyser through enzyme-linked immunosorbent assay method. Kit used was interleukin 6 human ELISA kit and sensitivity was less than 1 pg/ml. Normal levels were considered 1-7 pg/ml and any value above 7 pg/ml was considered raised.

Serum ferritin levels were measured by using VITROS 5600 integrated system which works by the principle of electrochemiluminescence immunoassay. The kit consisted of ready-to-use ferritin reagent, Elecsys Ferritin and CalSet. Normal range was considered to be 24 to 336 micrograms per liter and any value above 336 micrograms per liter was considered raised.

Serum lactate dehydrogenase was measured by the lactate kinetic method with a normal range of 140-280 U/L. Any value above 280 U/L was considered as raised.

Serum C-reactive protein was measured by immunoturbidimetry with normal range of 8-10 mg/L. Any value above 10 mg/l was considered raised.

D-Dimer was measured by automated latex enhanced immunoassay with normal range of -< or =500 ng/mL fibrinogen-equivalent units. Any value above 500 ng/ml fibrinogen-equivalent units was considered raised.

Study definition

Only the patients admitted to the COVID ICU were enrolled in the study. The criteria for admission in the COVID ICU were COVID-19 patients with oxygen saturation of less than 90% on room air or patients with respiratory rate of more than 30 breaths per minute on room air as per guidelines from the Indian Council of Medical Research and All India Institute of Medical Sciences.

Statistical analysis

Data were expressed by the measures of percentages (%), mean ± SD, or median and 25% to 75% inter-quartile range (IQR), as appropriate. All the Patients were divided into Cured group and non-survivors or Death group. The dichotomous variables were drafted by the use of thresholds which were relevant clinically. Variables which were Continuous and normally distributed were compared with t-test however Mann-Whitney U-test was used for non-normally distributed variables. ROC curves were constructed for mortality and ICU stay prediction. Correlation of COVID-PIRO score with the outcomes was done using ROC curves. Unpaired t-test was done for the effect of co-morbidities on COVID-PIRO score. SPSS software version.21 was used for analysis. All the statistical tests performed were two-tailed; p-value of < 0.05 was considered to be statistically significant.

## Results

A total of 240 patients were included in the analysis. Males were 72% and females were 28%. Mean age of participants was 60.27 ± 15.3 years. All the demographic and clinical characteristics are shown in Table 2.

**Table 2:** Demographic and clinical characteristics.

Character	Number of patients (n = 240)
Age	
18-40	30 (12.5%)
41-60	82 (34.1%)
>60	128 (53.3%)
Sex	
Male	172 (71.6%)
female	68 (28.3%)
Comorbidities	168 (70%)
Hypertension	87 (36.25%)
Diabetes mellitus	64 (26.6%)
Chronic obstructive pulmonary disease	24 (10%)
Ischemic heart disease	38 (15.8%)
Obesity	33 (13.75%)
Comorbidities absent	72 (30%)
25 Point Computed Tomography Severity Score	
<8	61 (25.4%)
9-15	100 (41.6%)
>15	79 (32.9%)
Blood pressure	
Systolic blood pressure < 90mmHg	117 (48.75%)
Systolic blood pressure > 90mmHg	123 (51.25%)
Heart rate	
>120 beats per minute	110 (45.8%)
<120 beats per minute	130 (54.1%)
Respiratory rate (with oxygen therapy)	
>20	235 (97.91%)
<20	5 (2.08%)
Multiple organ dysfunction syndrome	
Present	233 (97.08%)
Absent	7 (2.91%)
Laboratory profile	
COVID-PIRO score	14.4 ± 3
Intensive care unit stay (days)	9.09 ± 6.34
Blood urea (gm/dl)	60.2 ± 53.4
Serum creatinine (gm/dl)	1.8 ± 2.7
Serum glutamic-oxaloacetic transaminase (IU/dl)	100.8 ± 249
Serum glutamic pyruvic transaminase (IU/dl)	68 ± 170.4
Platelets	1.9 ± 1.1
Haemoglobin (gm/dl)	12.1 ± 2.5
Computed tomography severity score	12.6 ± 6.7
Reverse transcriptase polymerase chain reaction cycle length	15.7 ± 8.7

**Figure 2:**
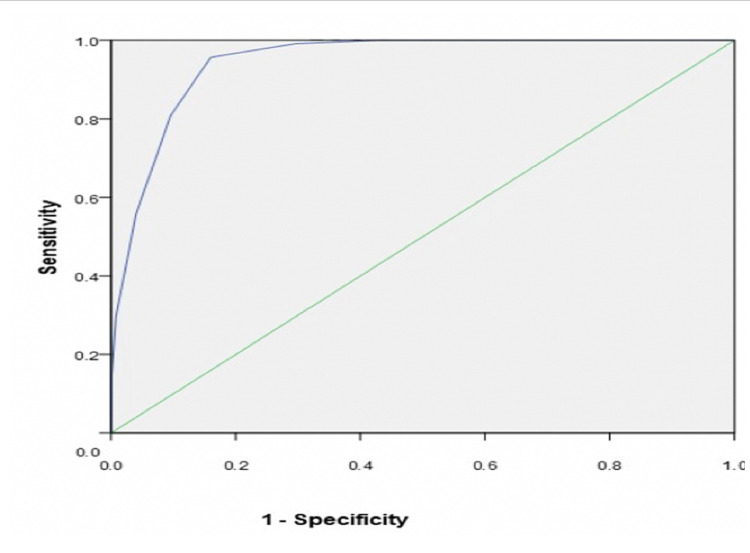
Area under curve showing mortality.

ROC curve analysis demonstrated that at a cut off of 14.5 COVID-PIRO Score showed sensitivity of 95% and specificity of 84% for mortality prediction (p < 0.001; AUC 0.946 ± SE 0.014; CI = 0.918 - 0.973) shown in Figure 2.

**Figure 3:**
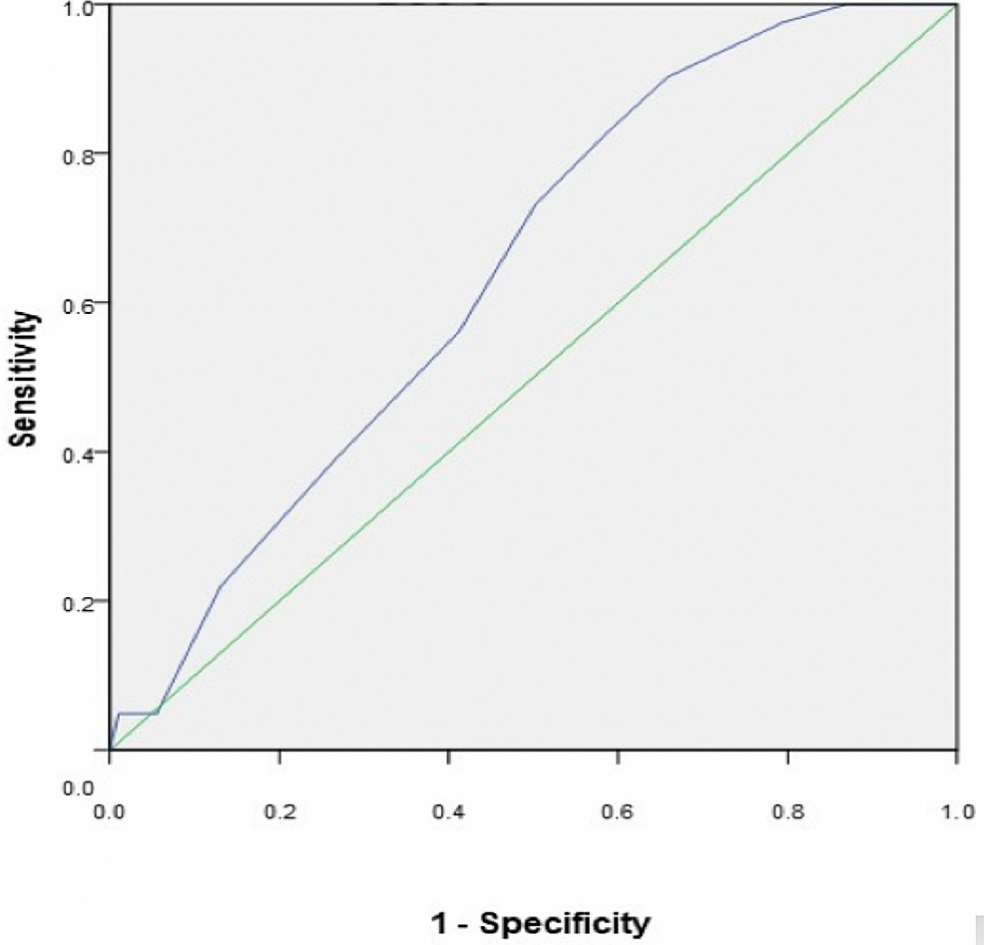
Area under curve showing hospital stay.

ROC curve analysis demonstrated that at a cut off COVID-PIRO Score of 15.5 having sensitivity of 56 % and specificity of 59 % for prediction of ICU stay (p < 0.01; AUC = 0.642 ± SE 0.042; CI = 0. 560 - 0.723) as shown in Figure 3.

Through the use of ROC curves, a cut of 14.5 COVID-PIRO score was derived. Outcomes, mortality prediction and length of ICU stay were analysed for COVID-PIRO score of less than and = 14.5 and more than 14.5. Table 3 shows out of 240 patients included in the study the group having PIRO ≤14.5 had a mortality rate of 1.25% as compared to the group having COVID-PIRO>14.5 which had mortality of 27.5%, with a discharge rate of 26.25% and 5%, respectively, in both groups (p = 0.0001).

**Table 3:** Correlation between PIRO score and outcome.

		Outcome		Total	א2-value
		Discharge	Death		
COVID-PIRO	≤14.5	105(26.25%)	5(1.25%)	110	153.06; p = 0.0001,S
	>14.5	20(5%)	110(27.5%)	130	
Total		125(31.25%)	115(28.75%)	240	

Multiple regression analysis was performed on the data considering COVID-PIRO to be the independent variable and age, gender, co-morbidities, CT-severity score, blood pressure, respiratory rate, pulse rate, RT-PCR cycle length, multi-organ dysfunction syndrome (MODS) and inflammatory markers as dependent variables to look for correlation between COVID-PIRO and various patient characteristics. Independent association of COVID-PIRO was found only with blood pressure as shown in Table 4.

**Table 4:** Multiple regression analysis.

Model		Unstandardized coefficients		Standardized coefficients	t	p-value	95.0% CI for B	
		B	Std. Error	Beta			Lower Bound	Upper Bound
M U L T I P L E ReH	PIRO	21.220	1.964					
	Age	0.005	0.009	0.025	0.555	0.580,NS	-0.012	0.022
	Gender	-0.017	0.280	-0.003	0.060	0.952,NS	-0.569	0.535
	Comorbidity	-0.356	0.288	-0.056	1.237	0.217,NS	-0.924	0.211
	Computed Tomography Severity Score	0.015	0.019	0.034	0.772	0.441,NS	-0.023	0.053
	Blood Pressure	-4.205	0.710	-0.714	5.923	0.0001,S	-5.604	-2.806
	Respiratory Rate	-0.007	0.025	-0.029	0.281	0.779,NS	-0.056	0.042
	Pulse Rate	0.002	0.002	0.051	1.034	0.302,NS	-0.002	0.006
	Reverse Transcriptase Polymerase Chain Reaction Cycle length	-0.011	0.028	-0.033	0.397	0.692,NS	-0.066	0.044
	Multiple Organ Dysfunction Syndrome	0.431	0.298	0.066	1.449	0.149,NS	-0.155	1.018
	Inflammatory Marker	-0.597	0.595	-0.044	1.004	0.317,NS	-1.769	0.575

## Discussion

This prospective observational study was undertaken in patients of COVID-19 admitted in intensive care units of a large tertiary care centre, identified with a group of variables associated with each component of the COVID-PIRO staging system independently associated with hospital mortality. 

CAPUCI study by Rello et al. had highlighted severity assessment scores based on the PIRO concept in patients with severe community-acquired pneumonia. They compared the PIRO score with the APACHE II score which performed better to identify patients with a higher risk of 28-day mortality [5]. Study by Lisboa et al. had calculated PIRO score in patients with ventilator-associated pneumonia, which also performed better than APACHE II score [6]. Few studies have attempted to develop a predictive or risk score model in COVID-19 patients [11]. PIRO score performed better than the SOFA score in severe sepsis and septic shock taking into account comorbidities and septic source as well as physiologic status. Mortality increased with increasing PIRO scores: PIRO < 5, (0%), PIRO 5 to 9, (5%) PIRO 10 to 14 (5%), PIRO 15 to 19 (37%) and PIRO ≥ 20 (80%) [10]. In our study, COVID-PIRO score > 14.5 showed increased mortality as well as hospital stays. Zou et al showed that older age, higher SOFA score, and elevated D-dimer at admission were risk factors for death of adult patients with COVID-19 [11]. Another study compared the severity of illness scores in ARDS patients with H1N1 infection and ARDS patients with COVID-19. They concluded that ARDS induced by COVID-19 had lower severity of illness scores at presentation and lower SOFA score-adjusted mortality [12-13].

Our results were superior to other scores in predicting mortality in COVID-19 patients [14,15]. Fan et al compared 7 scores in 654 COVID-19 patients with pneumonia. Among all seven scores that were determined by patients' information on admissions, all had different discrimination and variability as well sensitivity and specificity in predicting in-hospital death. In our study, with COVID-PIRO score having AUC 0.95; 95% CI 0.91 - 0.97 had better predictability in outcomes and hospital stays [11].In this study sensitivity (95%) and specificity (84%) of COVID PIRO > 14.5 were very high.

It is important to note that our study found COVID-PIRO Score to be associated independently with blood pressure. It has been previously shown in various studies and meta-analysis that hypertension as a co-morbidity increases mortality and severity of COVID19 [16]. This might be the reason behind blood pressure being significantly associated with COVID-PIRO Score.

Strength

This COVID-PIRO is very simple and more practical, so that it can be used in busy COVID ICUs as it is based on four easily assessable components. It represents a highly effective and easy to perform tool applicable for categorization and prognostication in ICUs.

Limitation

One of the limitations of this study was small sample size due to single-center being involved. Also, this scoring system needs validation in larger set of patients, is not widely used and lacks standardization.

## Conclusions

We conclude that COVID-PIRO score was highly useful model in predicting in-hospital mortality. It was useful in predicting ICU stay. Therefore, this model can prove to be a novel marker used in the ICU day in and out to help prognosticate ICU patients with COVID-19 infection. However, further studies are required in order to provide validation to this scoring system.
